# *O*-GlcNAc transferase couples MRE11 to transcriptionally active chromatin to suppress DNA damage

**DOI:** 10.1186/s12929-022-00795-1

**Published:** 2022-02-14

**Authors:** Aishwarya Gondane, Samuel Girmay, Alma Helevä, Satu Pallasaho, Massimo Loda, Harri M. Itkonen

**Affiliations:** 1 Department of Biochemistry and Developmental Biology, Faculty of Medicine, University of Helsinki, Helsinki, Finland; 2 Department of Pathology and Laboratory Medicine, Weill Cornell Medicine, New York-Presbyterian Hospital, New York, NY USA; 3 The Broad Institute of Harvard and MIT, Cambridge, MA USA; 4 The New York Genome Center, New York, NY USA

**Keywords:** DNA damage, Cyclin-dependent kinase 9, *O*-GlcNAc transferase, MRE11, Castration-resistant prostate cancer

## Abstract

**Background:**

Transcription, metabolism and DNA damage response are tightly regulated to preserve the genomic integrity, and *O*-GlcNAc transferase (OGT) is positioned to connect the three. Prostate cancer is the most common cancer in men, and androgen-ablation therapy halts disease progression. However, a significant number of prostate cancer patients develop resistance against anti-androgens, and this incurable disease is termed castration-resistant prostate cancer (CRPC). We have shown that combined inhibition of OGT and the transcription elongation kinase CDK9 induce CRPC-selective anti-proliferative effects. Here, we explain the functional basis for these combinatorial effects.

**Methods:**

We used comprehensive mass spectrometry profiling of short-term CDK9 inhibitor effects on *O*-GlcNAcylated proteins in an isogenic cell line system that models transition from PC to CRPC. In addition, we used both ChIP-seq and RNA-seq profiling, and pulldown experiments in multiple CRPC models. Finally, we validated our findings in prostate cancer patient samples.

**Results:**

Inhibition of CDK9 results in an OGT-dependent remodeling of the proteome in prostate cancer cells. More specifically, the activity of the DNA damage repair protein MRE11 is regulated in response to CDK9 inhibition in an OGT-dependent manner. MRE11 is enriched at the *O*-GlcNAc-marked loci. CDK9 inhibition does not decrease the expression of mRNAs whose genes are bound by both *O*-GlcNAc and MRE11. Combined inhibition of CDK9 and OGT or MRE11 further decreases RNA polymerase II activity, induces DNA damage signaling, and blocks the survival of prostate cancer cells. These effects are seen in CRPC cells but not in normal prostate cells. Mechanistically, OGT activity is required for MRE11 chromatin-loading in cells treated with CDK9 inhibitor. Finally, we show that MRE11 and *O*-GlcNAc are enriched at the prostate cancer-specific small nucleotide polymorphic sites, and the loss of MRE11 activity results in a hyper-mutator phenotype in patient tumors.

**Conclusions:**

Both OGT and MRE11 are essential for the repair of CDK9 inhibitor-induced DNA damage. Our study raises the possibility of targeting CDK9 to elicit DNA damage in CRPC setting as an adjuvant to other treatments.

**Graphical Abstract:**

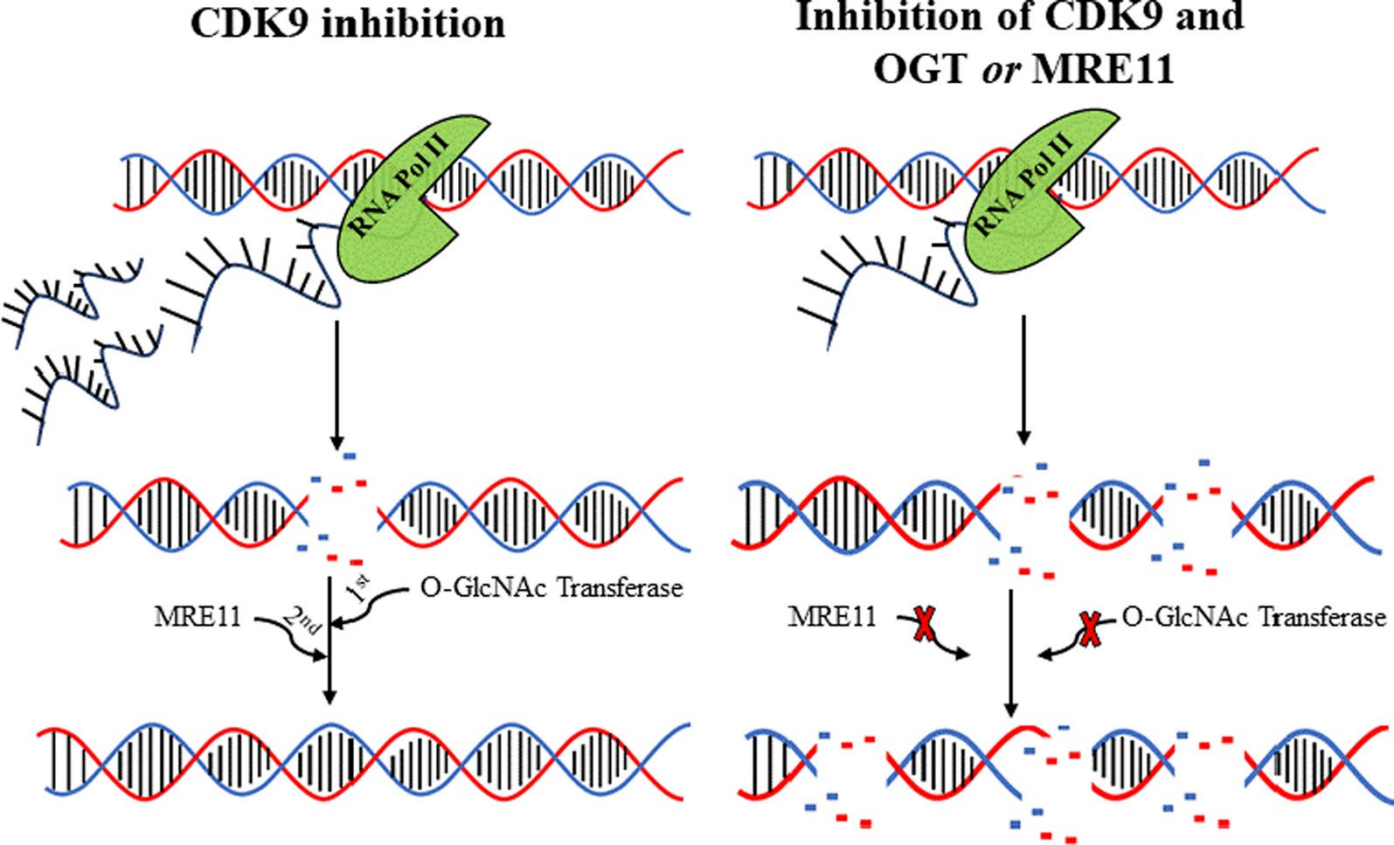

**Supplementary Information:**

The online version contains supplementary material available at 10.1186/s12929-022-00795-1.

## Background

Prostate cancer is the most common cancer in men and the disease is driven by the hyper-activated transcription factor, androgen receptor (AR) [41]. Androgen-ablation therapy halts disease progression; however, a significant number of prostate cancer patients develop resistance against AR-antagonists, and this incurable disease is termed castration-resistant prostate cancer (CRPC). Transcription machinery itself represents a point of vulnerability in prostate cancer.

AR-dependent transcriptional network is intimately coupled to DNA damage signaling. AR regulates the expression of the DNA damage repair genes [37], AR chromatin binding sites are highly mutated in prostate cancer cells [32], and activation of AR promotes generation of fusion genes [4]. Decreased AR activity downregulates the factors that protect cancer cells from DNA damage, including PARP proteins, which then become cancer cell-selective combinatorial lethal targets [28].

We have used AR-driven transcriptional networks to identify prostate cancer-relevant pathways. AR drives the expression of metabolic pathways to support high levels of *O*-GlcNAcylation in prostate cancer cells [17, 20, 33]. Nucleocytoplasmic *O*-GlcNAcylation is solely catalyzed by *O*-GlcNAc transferase (OGT), and this enzyme thereby functions as a major metabolic integration point [14, 24]. We have shown that OGT is overexpressed in aggressive prostate cancer [19], and we also discovered that OGT becomes a point of vulnerability during androgen-ablation [22]. AR-dependent transcription promotes *O*-GlcNAcylation in prostate cancer cells, and, reciprocally, many proteins of the transcription machinery are *O*-GlcNAcylated.

OGT is attracted to transcriptional start sites of many genes but the functional implications of this are not well understood. OGT and the catalytic product, *O*-GlcNAcylated proteins, are enriched on the promoters and overlap with RNA polymerase II (RNA Pol II) binding sites, but both, the enzyme and the product, have also been associated with the transcriptional repression [22, 39, 49]. Unexpectedly, however, inhibition of OGT has mild effects on the global transcription [7, 19, 22, 23, 46]. OGT modifies thousands of targets, and it is possible that the enzyme becomes a critical regulator of transcription only in the specifically stressed cells.

We performed an unbiased combinatorial lethality screen, which revealed that combined inhibition of OGT and cyclin-dependent kinase 9 (CDK9) is selectively toxic to prostate cancer cells [23]. CDK9 phosphorylates RNA Pol II to promote productive transcription elongation [8]. Functionally, OGT and CDK9 are linked at the level of transcription, but it is not inherently clear why OGT becomes essential when CDK9 activity is compromised. We hypothesized that OGT activity is remodeled in cells that are treated with CDK9 inhibitors.

Here, we report a comprehensive glycoproteomic profiling of prostate cancer cells treated with CDK9 inhibitor. We show that the activity of MRE11 is remodeled in response to CDK9 inhibition in an OGT-dependent manner. We go on to show that *O*-GlcNAcylation and MRE11 chromatin binding sites overlap, and that OGT activity is required for the MRE11 chromatin-loading. It has been previously established that MRE11 is required for initiation of the DNA double-strand break repair. Accordingly, we show that combined inhibition of CDK9 and either OGT or MRE11 induces robust DNA damage in prostate cancer cells. Finally, MRE11 and O-GlcNAc chromatin binding sites overlap with the common prostate cancer small nucleotide polymorphic-loci, and the loss of MRE11 activity is associated with the hyper-mutator phenotype in prostate cancer patient samples.

## Methods

### Cell culture, compounds and preparation of cell lysates

LNCaP, C4-2, 22RV1, and RWPE-1 cell lines were obtained from the American Tissue Culture Collection (ATCC). 22RV1 is a model of human CRPC, and the cell line was established from the androgen-dependent CWR22 xenograft after castration-induced regression and relapse [44]. Professor Stephen Plymate (University of Washington) kindly provided the LN95 cell line. LNCaP, C4-2, 22RV1 and PNT1 (the last from Sigma) were maintained in 10% fetal bovine serum (FBS) in RPMI medium. LN95 and RWPE-1 cells were maintained in 10% charcoal-stripped FBS-supplemented phenol red-free RPMI and keratinocyte-serum free media, respectively. The following compounds were obtained from MedChemExpress: YKL-5-124, NVP2, THZ531, Thiamet G and OSMI-4 [30]. Transfection of Ambion® Silencer Select siRNAs against CDK9 (s2834) and MRE11 (s8959) was achieved using RNAiMax and cells were collected after 4 days.

Cell lysates for western blotting were prepared as previously reported [19, 21]. In brief (all steps at + 4 °C), cells were washed with PBS, and incubated in cell lysis buffer supplemented with proteinase, phosphatase and *O*-GlcNAcase (Thiamet G) inhibitors for 15 min, centrifuged at 14,000 rpm for 5 min and the supernatant was collected. Protein concentration was determined with bicinchoninic acid (BCA) assay.

Lectin pulldowns [18] and cell fractionation [15] were performed as previously reported, except the cytoplasmic and nuclear fractions were combined here. For *O*-GlcNAc immunoprecipitation, we combined two antibodies: RL2 (Abcam: ab2739) and CTD110.6 (Cell Signaling Technologies: 9875), and used Pierce Direct IP Kit (ThermoFisher Scientific) according to manufacturer’s protocol to prepare the biological triplicate samples for mass spectrometry (MS). MS was performed by the Weill Cornell Medicine (WCM) Meyer Cancer Center Proteomics & Metabolomics Core Facility. The following antibodies were used to detect the proteins of interest, from Cell Signaling technology: p-Ser2-RNA Pol II (13499), OGT (24083) and CDK9 (2316); from Santa Cruz Biotechnology: MRE11 (135992), RNA Pol II (sc-56767) p-H2AX (517348) and H2AX (sc-517336); and from Abcam: RL2 (ab2739) and Actin (ab49900). Signal intensity of western blot (densitometry) was determined using Image Lab version 6.0 (Bio-Rad). RNA isolation was performed using the illustraMiniSpin-kit (GE Healthcare) according to manufacturer's instructions, cDNA was synthesized using the qScript cDNA Synthesis Kit (Quantabio) and the following primers were used in RT-qPCR: MYC F-TACCCTCTCAACGACAGCAG, R-TCTTGACATTCTCCTCGGTG and Actin F-TGGGACGACATGGAGAAAAT, R-AGAGGCGTACAGGGATAGCA.

### Proliferation assays

Incucyte live cell imaging system was used to measure the proliferation rate of cells according to manufacturer’s instructions (Sartorius). Crystal violet staining assay was used to assess the colony formation ability of the cells as previously reported [3]. RealTime-Glo® MT and CellTiter-Glo® 2.0 assays from Promega were used according to manufacturer’s instructions to measure the cell viability.

### Bioinformatics

Genome wide binding profiling of MRE11 after 15 min 5alpha-dihydrotestosterone (DHT) treatment and for *O*-GlcNAc were obtained from GSE63202 [38] and GSE121474 [22], respectively. UCSC Genome Browser was used to uplift the MRE11 ChIP-seq peaks called on HG18 to HG19. The ChIP-seq peaks were intersected using Venny 2.1, an online interactive tool. RNA-seq data for LNCaP cells treated with CDK9 and OGT inhibitors was obtained from GSE116778 [23]. The differentially expressed genes which were bound with MRE11 and/or *O*-GlcNAc were found using Microsoft Excel, and boxplots were made using R (version 4.1.1) and R studio (version 2021.09.0). Gene list of SNPs associated with prostate cancer were obtained from GCST006085 data deposited in GWAS catalog [43]. Upset plot was generated using UpSetR package in R [9].

## Results

### Inhibition of CDK9 activity induces selective hyper-glycosylation

We hypothesized that OGT activity is remodeled in response to CDK9 inhibition. First, we confirmed that combined inhibition of OGT and CDK9 selectively inhibits proliferation of prostate cancer cells but does not have effects on normal prostate cells, as previously reported [23] (Additional file 1: Fig. S1). OGT glycosylates thousands of proteins, and depleting its activity will affect a variety of processes: by identifying the factor OGT glycosylates to adaptively respond to CDK9 inhibition, we may be able to target that protein to establish a novel, more specific combinatorial treatment-strategy.

**Fig. 1:**
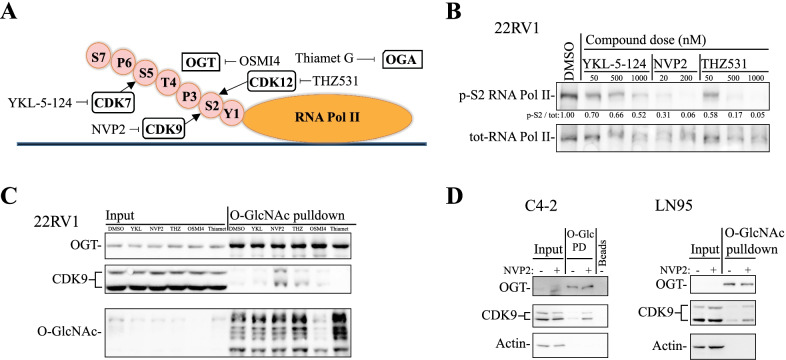
CDK9 inhibition remodels OGT activity. **A** C-terminal domain of RNA polymerase II is phosphorylated by cyclin-dependent kinase 7 (CDK7), CDK9 and CDK12 to promote transcription initiation, elongation and to keep up elongation of longer genes, respectively. Small molecule inhibitors: YKL-5-124, NVP2, THZ531, OSMI-4 and Thiamet G target CDK7, CDK9, CDK12, OGT and OGA, respectively. **B** To identify doses affecting RNA Pol II phosphorylation, cells were treated with increasing doses of specific inhibitors for 4 h and analyzed using western blot. Densitometry was used to measure the signal intensity. **C** 22RV1 cells were treated with CDK7, CDK9, CDK12, OGT and OGA inhibitor for four hours, after which samples were subjected to wheat-germ agglutinin-lectin pulldown and analyzed using western blot. **D** CRPC cell lines were treated with CDK9 inhibitor NVP2 for 4 h and the *O*-GlcNAcylated proteins were enriched using lectin pulldowns (*O*-Glc PD); the samples were analyzed using western blot

To probe the possibility that CKD9 inhibition induces selective remodeling of OGT activity, we need to include the other major transcriptional kinases as controls. RNA polymerase II (RNA Pol II) is phosphorylated predominantly by CDKs 7, 9 and 12/13 at the specific stages of transcription initiation, release for productive elongation and maintenance of phosphorylation in the long genes (Fig. 1A). First, we treated prostate cancer cells for four hours with increasing doses of the highly specific inhibitor against each kinase, to establish doses that robustly decrease RNA Pol II phosphorylation: 500 nM for CDK7 and CDK12/13 inhibitors and 20 nM for CDK9 inhibitor were found to achieve this (Fig. 1B).

We hypothesized that CDK9 inhibition affects *O*-GlcNAcylation of CDK9 because combined inhibition of OGT and CDK9 is toxic to prostate cancer cells [23], OGT inhibition decreases phosphorylation of CDK9 [46] and CDK9 is known to be *O*-GlcNAcylated [13]. To assess the effects on CDK9 *O*-GlcNAcylation, we performed *O*-GlcNAc pulldown experiments. Inhibition of CDK9 activity induced a robust *O*-GlcNAcylation of itself. Similar, albeit less prominent effect was also observed for the inhibition of the elongation kinases CDK12/CDK13, which also increased *O*-GlcNAcylation of CDK9 (Fig. 1C). We confirmed that inhibition of CDK9 activity increases *O*-GlcNAcylation of itself in two additional models of CRPC, LN95 and C4-2 cells (Fig. 1D). There are two CDK9 isoforms expressed in normal prostate and CRPC cells (Additional file 1: Fig. S2A). Both of these isoforms are targeted by NVP2 [35]. OGT modifies itself, which we used a positive control to confirm the pulldown efficiency after every treatment. As expected, OGT and OGA inhibitors depleted and increased global *O*-GlcNAcylation, respectively (Fig. 1C). Unexpectedly, neither OGT nor OGA inhibitor affected *O*-GlcNAcylation of CDK9 (Fig. 1C). In contrast, inhibition of the transcription elongation kinases (CDK9 and CDK12) increased *O*-GlcNAcylation of CDK9. Based on these data, we propose that the *O*-GlcNAcylation of CDK9 is not a major mechanism regulating CDK9 in the unstressed cells, but becomes important in the cells experiencing transcriptional stress.

To summarize, targeting CDK9 directs OGT to glycosylate CDK9, but we did not yet know if CDK9 inhibitor-induced remodeling of the OGT activity is more widespread.

### Characterization of CDK9 inhibitor-induced glycoproteome using mass spectrometry

Proteins that get hyper-glycosylated when CDK9 activity is depleted can explain the mechanistic basis for the combinatorial lethality between compounds targeting OGT and CDK9. We hypothesized that OGT activity is remodeled in response to CDK9 inhibitor treatment because combined inhibition of OGT and CDK9 is toxic to prostate cancer cells [23], OGT activity is remodeled in response to stress [25, 31] and OGT-dependent glycosylation alters protein function [24, 34]. By identifying the proteins that are increasingly *O*-GlcNAcylated in response to CDK9 inhibition, we can establish mechanistically, why combined inhibition of OGT and CDK9 is toxic to prostate cancer cells. To identify the proteins, we performed immunoprecipitation with *O*-GlcNAc-specific antibodies followed by mass spectrometry. For these experiments, we used two cell lines: LNCaP (PC) and LN95 (CRPC). LN95 was established through an extended androgen-deprivation of the LNCaP cells, and is a model of CRPC that grows in the absence of androgens. These cell lines enable us to discover common and shared responses in an isogenic cell line pair that models the development of CRPC.

**Fig. 2:**
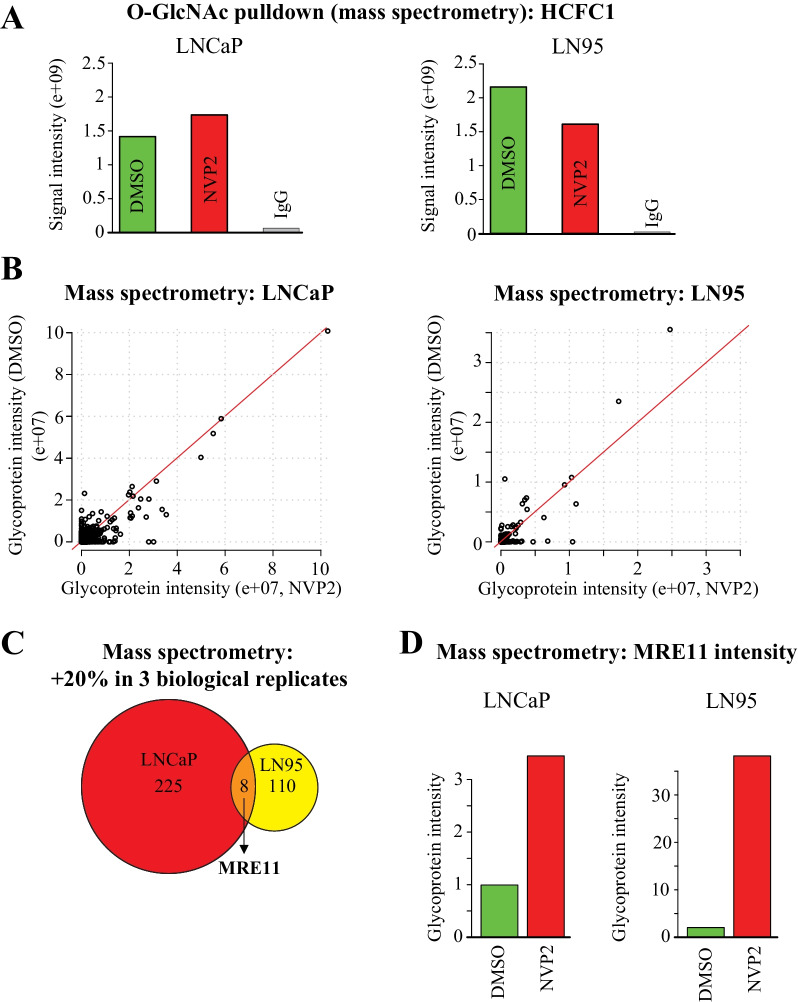
CDK9 inhibition increases *O*-GlcNAcylation of MRE11. **A** HCFC1 was used as a positive control to confirm the efficiency of immunoprecipitation. LNCaP and LN95 cells were treated with CDK9 inhibitor for 4 h and the *O*-GlcNAcylated proteins were enriched using RL2 and CTD110.6 antibodies, followed by mass spectrometry analysis. The data presented are average of three biological replicates (signal intensity). **B** Scatter plots showing the *O*-GlcNAcylated proteins. The signal intensity values represented here were normalized to IgG. **C** Venn diagrams showing the proteins that had > 20% increased *O*-GlcNAcylation as compared to DMSO in all three biological replicates: we considered these as the hyper-*O*-GlcNAcylated proteins. **D** Bar plots indicating the *O*-GlcNAcylation intensity of MRE11 in LNCaP and LN95 cells. The values are average of three biological replicates. First, values were normalized to negative immunoprecipitation, and second, data are presented relative to control (DMSO), which was set to value of one

We performed biological triplicate mass spectrometry experiments in LNCaP and LN95 cell lines after four hours treatment with CDK9 inhibitor. As expected, we detected a robust enrichment of a known hyper-*O*-GlcNAcylated protein HCFC1 [24]; its *O*-GlcNAcylation was not affected by CDK9 inhibition (Fig. 2A). In addition, CDK9 was inducibly *O*-GlcNAcylated in response to CDK9 inhibition in both LNCaP and LN95 cell lines (Additional file 1: Fig. S2B). Most of the *O*-GlcNAc-proteome remained unchanged in both cell lines (Fig. 2B).

Interestingly, we noted that a small sub-set of proteins (eight) is increasingly *O*-GlcNAcylated in both cell lines (Fig. 2C). Because combined inhibition of CDK9 and OGT is toxic to both PC and CRPC cells [23], we hypothesized that the proteins, which are increasingly *O*-GlcNAcylated in both cell lines, are the most likely candidates to explain why OGT activity becomes important when CDK9 is inhibited. We focused on the DNA damage response protein MRE11 (Fig. 2D) because this protein was the only one that is directly druggable [11], it is not previously known to be regulated by OGT, it is overexpressed in the aggressive prostate cancer [47], and, as discussed in the introduction, DNA damage response is remodeled in the CRPC [42]. MRE11 is an endonuclease that is required for the initiation of the DNA double-strand break repair and removal of the protein blocks from the chromatin [36]. In lectin-based validation experiments, we did not detect a robust increase in MRE11 *O*-GlcNAcylation in response to CDK9 inhibitor treatment (Additional file 1: Fig. S2C). We therefore hypothesize that MRE11 operates in loci that are hyper-*O*-GlcNAcylated, and OGT activity becomes important to regulate MRE11 in response to CDK9 inhibitor treatment.

### *O*-GlcNAc and MRE11 are enriched on the genes whose expression resist CDK9 inhibition

**Fig. 3:**
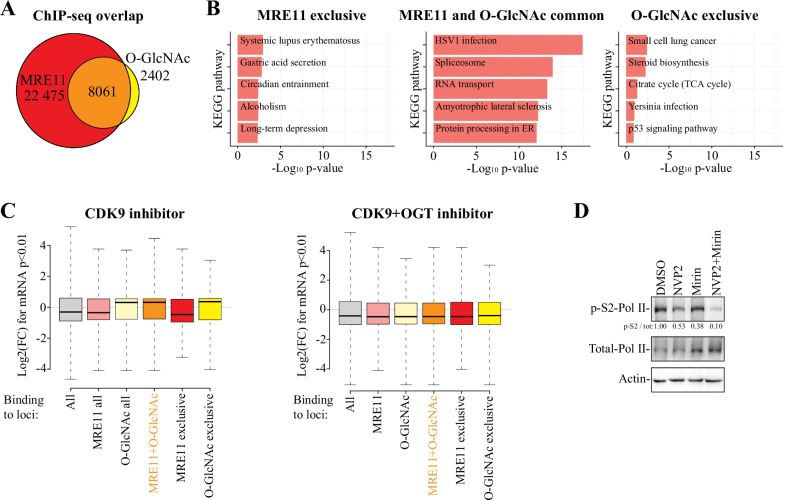
MRE11 and *O*-GlcNAc marked genes resist CDK9 inhibitor effect on mRNA levels. **A** Venn-diagram showing the overlap between *O*-GlcNAc (GSE121474) and MRE11 (GSE63202) ChIP-seq peaks from LNCaP cells. **B** KEGG pathway enrichment analysis of the indicated gene-sets as reported in **A**. **C** Effects of CDK9 and CDK9 + OGT inhibition on expression levels of genes bound by MRE11 and/or *O*-GlcNAc, or neither. Significantly (p < 0.01) affected mRNAs after four hours treatment of LNCaP cells were identified from GSE116778. **D** C4-2 cells were treated with 20 nM NVP2, 10 µM Mirin and combination of both compounds for 8 h. The effects on RNA Pol II phosphorylation was assessed by western blot. Data is representative of four biological replicates

We used chromatin immunoprecipitation coupled to massively parallel sequencing (ChIP-seq) to assess if MRE11 and *O*-GlcNAcylation are enriched at the same loci. Strikingly, 80% of the 10,000 *O*-GlcNAc peaks overlapped with the MRE11 binding sites (Fig. 3A). Pathway enrichment analysis of the genes marked by both *O*-GlcNAc and MRE11 revealed a highly significant enrichment of processes related to RNA metabolism (spliceosome and RNA transport, Fig. 3B). In contrast, genes marked exclusively by either MRE11 or *O*-GlcNAc were not as significantly enriched for any particular process. Next, we assessed if the genes marked by *O*-GlcNAc, MRE11 or *O*-GlcNAc and MRE11 are affected by the CDK9 inhibitor treatment using AT7519-compound. As expected, most mRNAs are downregulated when cells are treated with CDK9 inhibitor (Fig. 3C). Interestingly, the genes marked by both MRE11 and *O*-GlcNAc resist the effects of CDK9 inhibitor, while the genes marked by MRE11 only (22 475 genes) are modestly more sensitive to CDK9 inhibitor treatment (Fig. 3C and Additional file 1: Fig. S3). When we combined CDK9 inhibitor with OGT inhibitor, the fraction of the upregulated genes was no longer detected (Fig. 3C). Pathway analysis of the genes marked by *O*-GlcNAc and *O*-GlcNAc + MRE11 and that were upregulated in response to CDK9 inhibitor treatment, were highly enriched for a single pathway, spliceosome (Additional file 1: Fig. S4), in accordance to our previous report [16]. Based on these data, MRE11 activity is important to maintain RNA Pol II activity when cells are treated with the CDK9 inhibitor. Indeed, combined targeting of CDK9 and MRE11 (using Mirin [11]), further suppressed RNA Pol II phosphorylation (Fig. 3D).

To summarize our finding so far, chromatin binding sites of *O*-GlcNAc and MRE11 overlap, and genes marked by the two resist CDK9 inhibitor effects at the mRNA level. MRE11 has been previously shown to be enriched on the highly transcribing genes [40]. However, it is not known if OGT activity affects MRE11 function.

### Combined inhibition of CDK9 and either OGT or MRE11 induces DNA damage

**Fig. 4:**
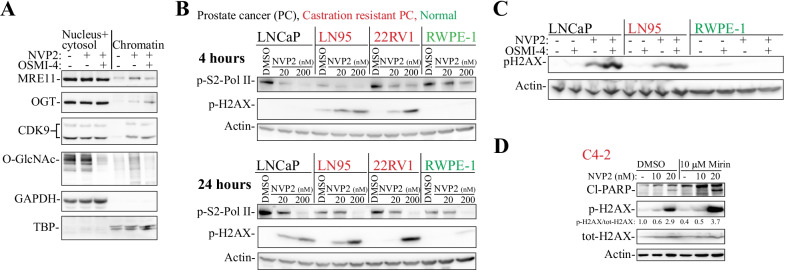
CDK9 inhibition induces DNA damage in prostate cancer cells. **A** C4-2 cell line was treated with 20 nM NVP2 or 20 nM NVP2 + 20 µM OSMI-4 for four hours, fractions isolated and samples analyzed using western blot. **B** Inhibition of CDK9 using NVP2 induces DNA damage in prostate cancer cells as measured using phosphorylation of H2AX. **C**, **D** Cells were treated for 24 h and analyzed using western blot

We hypothesized that OGT is important for chromatin-association of MRE11. It has been previously established that OGT activity is required for the RNA Pol II promoter entry [27]. To test if OGT regulates MRE11 chromatin-association, we isolated nuclear/cytosolic- and chromatin-fractions from prostate cancer cells treated with CDK9 inhibitor in the presence and absence of the OGT inhibitor OSMI-4. CDK9 inhibitor treatment increased chromatin binding of MRE11 and this effect was antagonized when cells were simultaneously treated with CDK9 and OGT inhibitors (Fig. 4A). We also noted that CDK9 inhibitor increased chromatin-loading of OGT and this resulted in an increased O-GlcNAcylation of the chromatin-associated proteins but not nuclear/cytosolic proteins, and the effects on O-GlcNAcylation were reversed in cells treated with the OGT inhibitor. Finally, CDK9 chromatin-association was increased when cells were treated with the CDK9 inhibitor but this response was not dependent on OGT.

MRE11 is required for the repair of double-strand breaks, and we therefore hypothesized that targeting CDK9 activates DNA damage signaling. Indeed, treatment of prostate cancer cells with the highly specific CDK9 inhibitor NVP2, or a structurally unrelated CDK9 inhibitor AT7519, induced accumulation of the DNA damage response marker p-H2AX (Fig. 4B and Additional file 1: Fig. S5A). Importantly, CDK9 inhibition did not cause the activation of the DNA damage marker in a model of normal prostate cells (RWPE-1, Fig. 4B and Additional file 1: Fig. S5B). We confirmed that CDK9 inhibitor doses that induce selective DNA damage also decrease RNA Pol II activity in PC, CRPC and normal cells by measuring MYC mRNA levels after 4 h treatment (Additional file 1: Fig. S5C).

**Fig. 5:**
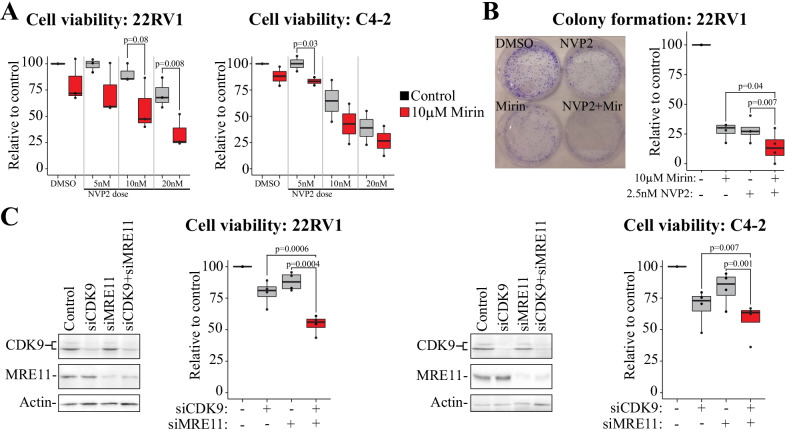
Co-targeting of CDK9 and MRE11 is toxic to prostate cancer cells. Data presented is always from at least three biological replicates and Student’s t-test was used to assess the statistical significance. **A** Combined inhibition of CDK9 and MRE11 combinatorically inhibits proliferation of CRPC cells (C4-2 and 22RV1) as determined using cell viability assay. **B** Combined inhibition of CDK9 and MRE11 blocks the colony-formation of 22RV1 cells. Cells were treated with 2.5 nM NVP2 either in the presence or absence of 10 µM Mirin. Left: example of colony-formation after seven days. Right: To get the average and SEM of four biological replicates, crystal violet dye was extracted and absorbance measured. Student’s t-test was used to assess the statistical significance. **C** Knockdown (KD) of both CDK9 and MRE11 significantly suppresses proliferation of CRPC cells. KD was performed for 4 days after which western blot was used to confirm silencing, and viability assays were used to evaluate effects on proliferation

Targeting either OGT or MRE11 enhances CDK9 inhibitor-induced DNA damage. Because inhibition of CDK9 induces DNA damage and OGT is required for MRE11 chromatin-loading, combined inhibition of CDK9 and either OGT, or MRE11 directly, should compromise the DNA damage response. Indeed, combined treatment of prostate cancer cells with OGT inhibitor and the CDK9 inhibitor NVP2 induced the accumulation of p-H2AX in PC and CRPC cells but not in the normal prostate cells (Fig. 4C). Similarly, treatment of CRPC cells in combination with CDK9 and MRE11 inhibitors induced accumulation of the DNA damage marker p-H2AX (Fig. 4D and Additional file 1: Fig. S5D). Next, we used proliferation assays to evaluate if targeting MRE11 sensitizes CRPC cells to CDK9 inhibition. Indeed, MRE11 inhibition significantly potentiated the anti-proliferative effects of CDK9 inhibitor on CRPC cell lines C4-2 and 22RV1, as measured using cell viability and crystal violet assays (Fig. 5A and Additional file 1: Fig. S6). In addition, the combination also blocked the colony-forming ability of the CRPC cells (Fig. 5B). Finally, simultaneous knockdown of both CDK9 and MRE11 significantly decreased both 22RV1 and C4-2 cell survival when compared to knockdown of either alone (Fig. 5C).

Based on these data, OGT activity is required for MRE11 chromatin-loading to activate the pro-survival DNA damage repair in response to CDK9 inhibitor treatment. Our data propose that the loss of MRE11 activity should be synthetically lethal with CDK9 inhibitors, and in this context, it becomes important to asses if such losses exist in prostate tumors.

### MRE11 truncating mutations associate with high mutation burden and CRPC-specific SNPs

**Fig. 6:**
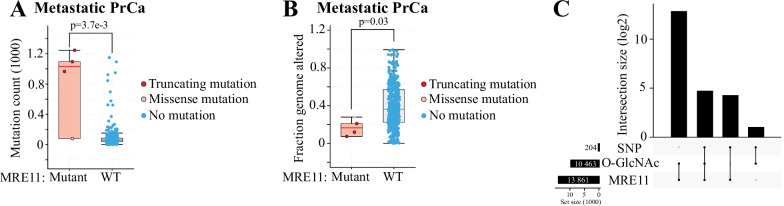
MRE11 is enriched on prostate cancer-specific mutation hotspots (small nucleotide polymorphisms). **A** Bar plots depicting mutational burden and **B** percentage of genome affected by the copy number gains or losses in MRE11 mutant and wild type prostate cancer patient samples. The plot was generated using cBioPortal and dataset Abida et al. (2019) [1]. **C)** Upset plot showing the overlap (at the level of gene-loci) between the previously published prostate cancer specific SNPs (GCST006085), and ChIP-seq data for *O-*GlcNAc (GSE121474) and for MRE11 (GSE63202)

We hypothesized that deletions or mutations in the MRE11 gene are associated with a specific mutational signature. In general, MRE11 expression is increased in prostate cancer [47]. Interestingly though, MRE11 gene has truncating mutations in a subset of metastatic prostate cancer patient samples, and any of these truncating mutations are associated with a hyper-mutator phenotype of the tumor (Fig. 6A). This mutational signature is reminiscent of the mismatch repair (MMR) deficiency, and these same patients had significantly less of copy number gains or losses when compared to MRE11 wild-type samples (Fig. 6B). MRE11 truncating mutations increase mutational burden with higher penetrance than alterations in the DNA repair genes CDK12, CHEK2 and ATM (Additional file 1: Fig. S7). Inactivating mutations in CDK12, CHEK2 and ATM are biomarkers of DNA repair deficiency in a clinical trial (NCT05011383). Prostate cancers are immunologically cold, and identification of markers that would predict sensitivity to immune checkpoint blockade are of high interest. High mutation burden due to the loss of MMR has been established as a predictive biomarker of sensitivity to immune checkpoint blockade [26]. Tumors with MRE11 truncating mutations may also be susceptible to immunotherapies. To conclude, MRE11 mutations are rare in prostate cancer tumors, but appear to cause a specific molecular signature.

Based on the data presented in this manuscript and the existing literature, both MRE11 and O-GlcNAc are enriched on the actively transcribing genes to protect these genes from DNA damage. Occasionally, MRE11 fails in inducing the appropriate DNA damage repair, which should result in small nucleotide polymorphisms (SNPs). Indeed, the majority of the high-confidence prostate cancer specific SNPs are bound by either MRE11 or O-GlcNAc or both (Fig. 6C).

## Discussion

Here, we identify MRE11 as a protein whose activity is regulated by OGT in response to CDK9 inhibitor-induced DNA damage. We earlier performed a comprehensive screen to discover compounds that sensitize prostate cancer cells to OGT inhibitors, and this work led to the discovery of combinatorial lethal interaction between OGT and CDK9 inhibitors that is specific to prostate cancer cells [23]. Here, we showed that this interaction is explained to large extend due to the CDK9 inhibitor-induced DNA damage, which is activated only in the prostate cancer but not in normal prostate cells (Fig. 4B). The appropriate repair of CDK9 inhibitor-induced DNA damage depends on both OGT and MRE11 (Fig. 4A, C and D). There are no clinical-grade OGT or MRE11 inhibitors, but we believe that our findings can be used to better understand the success of CDK9 inhibitors in clinical trials, and also to identify the right patients to receive these compounds. For example, the ability of CDK9 inhibitors to sensitize cancer cells to traditional chemotherapy warrants investigation.

It is fascinating how depleting the activity of the seemingly essential transcriptional kinase, CDK9, is tolerated by the normal cells but not the cancer cells. CDK9 inhibitors have gained significant success in clinical trials against myeloid cancers, and these compounds have also been trialed in the solid malignancies [8, 12].

The anti-cancer activity of the CDK9 inhibitors may be explained through a more complex mechanism than simply decrease in transcription. Cancer cells typically have compromised DNA damage response, which would selectively sensitize these malignant cells and save the normal cells from CDK9 inhibitors. However, this is essentially why chemotherapy works, and the clinical success of the CDK9 inhibitors is likely explained through even more complex mechanism.

Transcription-associated DNA damage is enriched at the transcription start sites of the highly expressed genes in a given cell type. In normal cells, the highly transcribed genes have significantly higher rate of the double-strand breaks (DSB) than the lowly transcribed genes [29, 48]. Particularly the genes that have high levels of RNA Pol II pause-release, have high levels of DSBs [10]. CDK9 inhibition is known to cause RNA Pol II accumulation on the transcription start sites [2]. Here, we show that CDK9 inhibition causes OGT-dependent chromatin-loading of MRE11 (Fig. 4A), and that depletion of either OGT or MRE11 activities enhances CDK9 inhibitor-induced DNA damage (Fig. 4C and D). We propose that targeting CDK9 causes DNA damage, which, if unrepaired, leads to mutations.

Prostate cancer specific SNPs are bound by MRE11 and O-GlcNAc at different levels, which we propose to represent the temporal separation in the chromatin-arrival of OGT and MRE11 during DNA repair. We show that in response to CDK9 inhibitor-induced DNA damage, OGT arrives to chromatin to increase chromatin O-GlcNAcylation (Fig. 4A). Chen and Yu (2016) showed that OGT is recruited to sites of DNA damage to increase the local O-GlcNAcylation in the vicinity of the damaged DNA [6]. Based on our experiments, OGT activity is required for the chromatin-loading of MRE11. In prostate cancer specific SNPs, most of the loci are marked either by MRE11, O-GlcNAc or both, which likely represents the temporal separation in the chromatin-arrival of OGT and MRE11 during the DNA repair-process (Fig. 6C).

MRE11 appears to be largely dispensable for the unperturbed transcription in prostate cancer cells because inhibition of MRE11 did not decrease RNA Pol II phosphorylation. In contrast, upon transcriptional stress that we achieved through CDK9 inhibitor treatment, MRE11 was required to restore RNA Pol II phosphorylation and to maintain genome integrity. This is of a particularly high interest in prostate cancer, because the supraphysiological activation of the prostate cancer-relevant transcription factor, androgen receptor, induces DNA damage in prostate cancer cells [5]. Intermittent high dose testosterone is currently evaluated in a Phase II clinical trial (NCT05011383) in the patients whose tumors present DNA repair deficiency (alterations in the genes ATM, CDK12 or CHEK2). Mutations in these DNA repair factors do not cause as significant hyper-mutator phenotype as do MRE11 mutations (Fig. 6A and Additional file 1: Fig. S7). We propose that inactivation of MRE11 is a biomarker of sensitivity to supraphysiological androgen-therapy.

Increased mutational burden has the potential to attract the immune system to the malignant cells. We observed that CDK9 inhibitors rapidly induce DNA damage signaling that persists after one day of the treatment (Fig. 4B). We did not detect activation of a robust DNA damage signaling in the normal cells in these same conditions. Cancer cell-selective induction of the excessive DNA damage in response to CDK9 inhibitor treatment is likely to cause mutations in the malignant cells. This is of particular interest in the CRPC, because the prostate tumors are poorly immunogenic [45]. In the future, the ability of CDK9 inhibitors to boost the immune response against the CRPC cells should be explored in more detail.

## Conclusions

To conclude, here we show that OGT activity is required for the chromatin-loading of MRE11, and in turn, MRE11 activity is required to activate the appropriate DNA damage signaling in response to CDK9 inhibition. Truncating mutations of the MRE11 gene are significantly associated with the hyper-mutator phenotype in patient samples, and our data proposes that MRE11 mutant tumors would be sensitive against the CDK9 inhibitors. Finally, our data link OGT and MRE11 to control RNA Pol II release for transcription elongation.

## Supplementary Information

**Additional file 1:** Supplementary figures and supplementary figure legends.

## Data Availability

All the data and materials are described in the appropriate sections and provide citations to the external databases/publications.

## Abbreviations

MRE11: MRE11 homolog, double strand break repair nuclease
AR: Androgen receptor
CRPC: Castration-resistant prostate cancer
CDK9: Cyclin-dependent kinase 9
DSB: Double-strand breaks
FBS: Fetal bovine serum
MMR: Mismatch repair
MS: Mass spectrometry
OGT: *O*-GlcNAc transferase
RNA Pol II: RNA polymerase II
