# Supplementary material for: O-GlcNAc transferase couples MRE11 to transcriptionally active chromatin to suppress DNA damage
Source: J Biomed Sci. 2022 Feb 14;29:13. doi: 10.1186/s12929-022-00795-1 (PMC8842528; doi:10.1186/s12929-022-00795-1)
Supplement: Supplementary file 1 — Additional file 1: Supplementary figures and supplementary figure legends. [file 12929_2022_795_MOESM1_ESM.pdf]

Supplementary figures legends

Models of prostate cancer

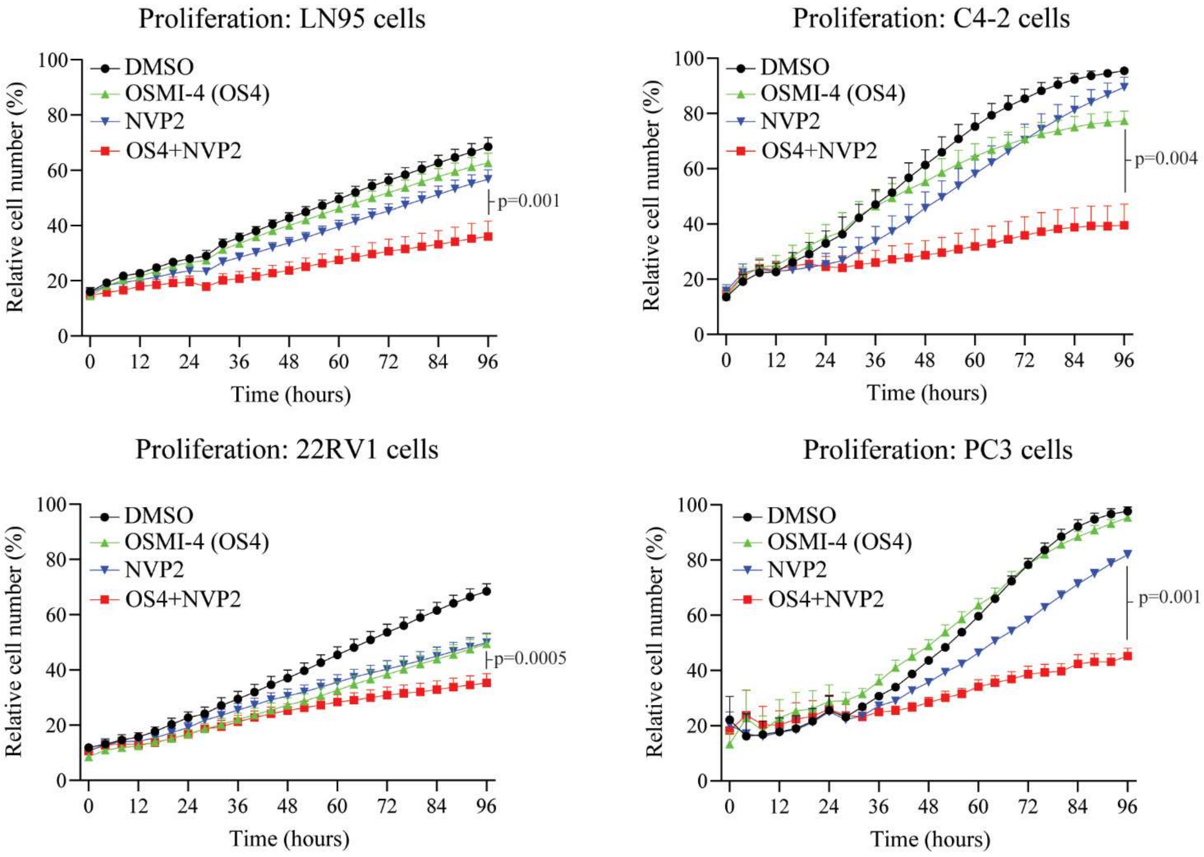

Models of normal prostate cells

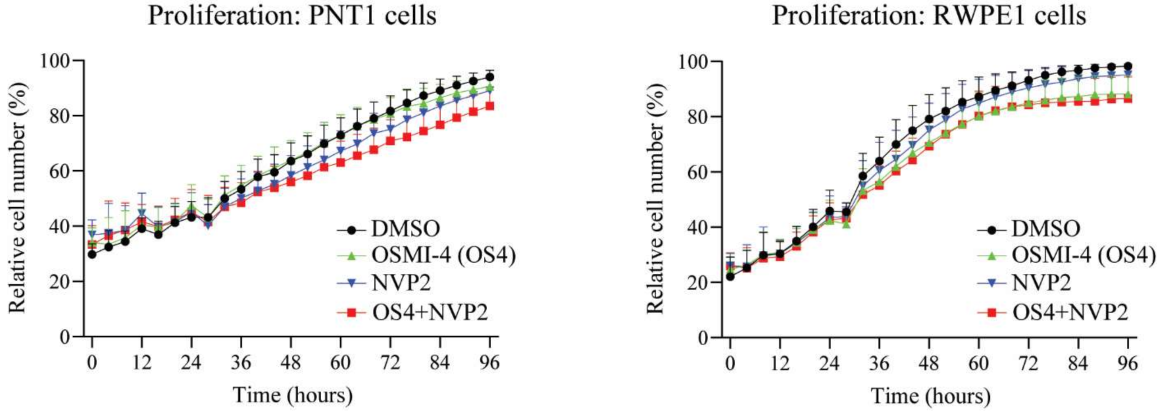

Supplementary Figure 1. Combined inhibition of CDK9 and OGT selectively inhibits the proliferation of prostate cancer cells but not normal prostate cells. Models of prostate cancer: LN95, C4-2, 22RV1, and PC3 or normal prostate cells (PNT1 and RWPE-1) were treated with CDK9 inhibitor (10nM NVP2) either in the presence or absence of OGT inhibitor OSMI-4 (20μM) for 96 hours to assess cell proliferation rate over time using Incucyte. Statistical analysis was performed using student's t-test. The data shown is an average of four biological replicates with SEM.

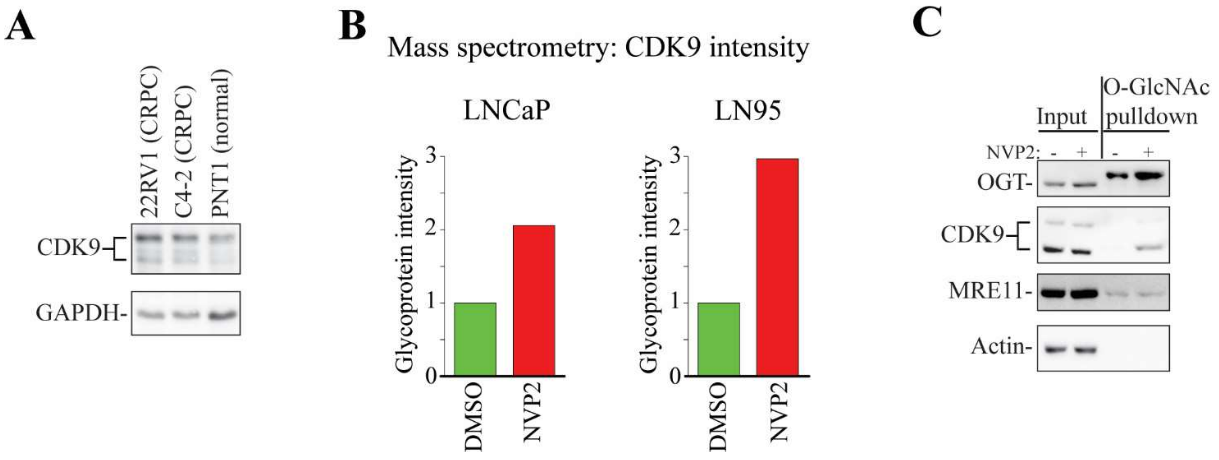

Supplementary Figure 2. CDK9 expression and O-GlcNAcylation of CDK9 and MRE11. A) The expression of CDK9 in CRPC and normal prostate cells was evaluated using western blot. Data is representative of two biological replicates. **B)** Cells were treated with 20nM NVP2 for 4 hours, O-GlcNAcylated proteins immunoprecipitated and samples were analyzed using mass spectrometry. The values are average of three biological replicates of the mass spectrometry data reported in this manuscript. First, values were normalized to negative immunoprecipitation (IgG). Second, data are presented relative to control (DMSO), which was set to value of one. **C)** Cells were treated with 20nM NVP2 for 4 hours and lectin pulldown was used to evaluate changes in O-GlcNAcylation of the selected proteins. Data is representative of two biological replicates.

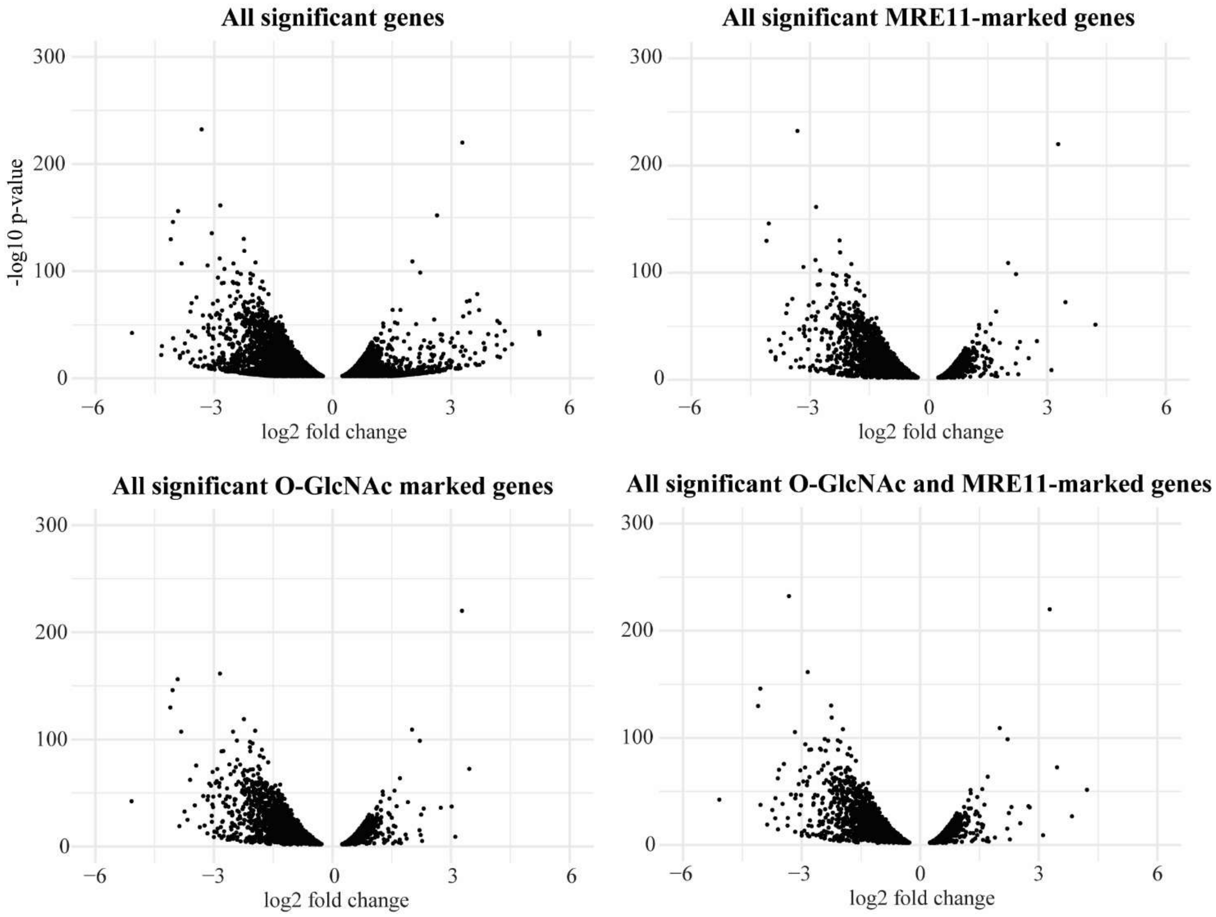

Supplementary Figure 3. The effect of CDK9 inhibitor AT7519 on gene expression. LNCaP cells were treated with 0.5µM AT7519 for four hours and analyzed by RNA-seq (GSE116778). Presented are significantly affected mRNAs ($p<0.01$).

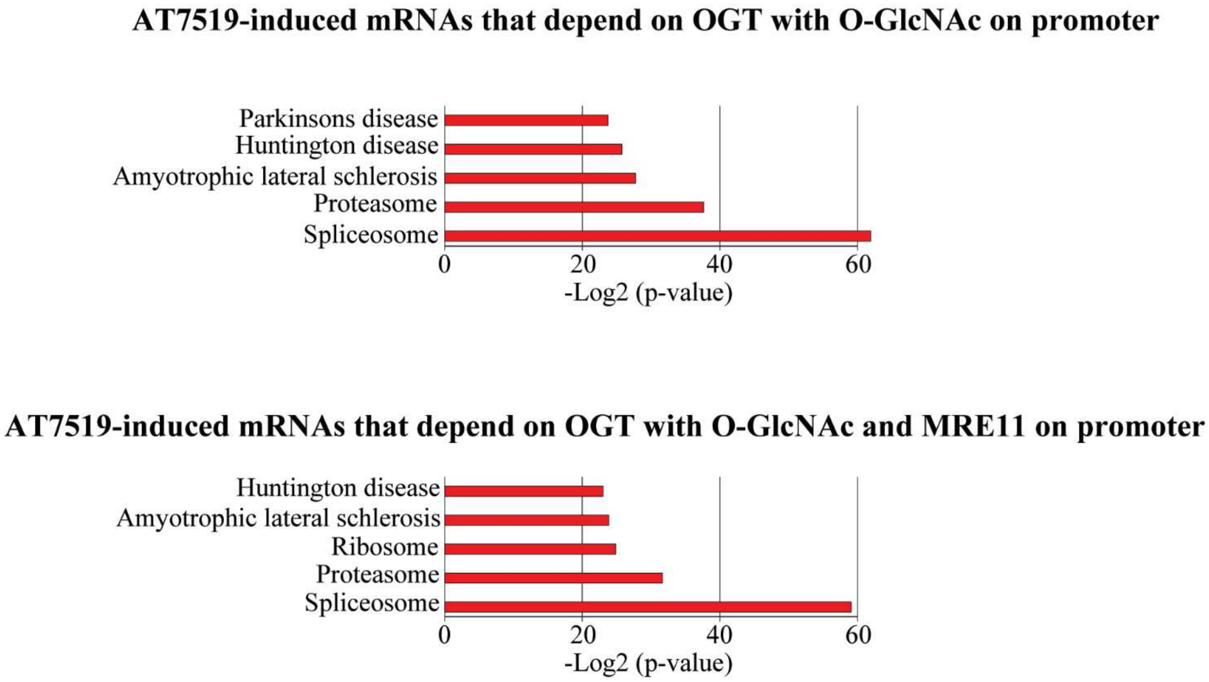

Supplementary Figure 4. CDK9 inhibition upregulates the expression of genes related to spliceosome. Pathway enrichment analysis using Enrichr (KEGG) [2]. Genes were selected based on the following criteria: significantly ($p<0.01$) upregulated in response to CDK9 inhibitor treatment (but not if CDK9 inhibitor was combined with the OGT inhibitor) and bound by O-GlcNAc (**top panel**) and O-GlcNAc+MRE11 (**bottom panel**).

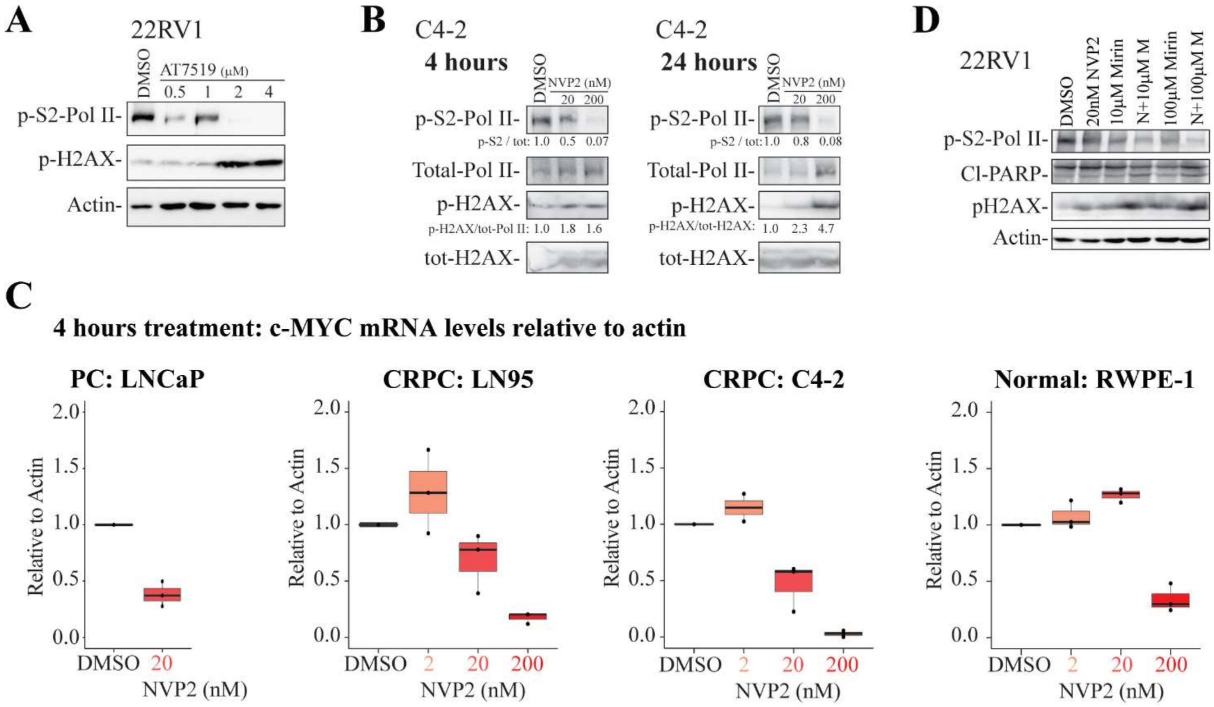

Supplementary Figure 5. Targeting CDK9 and CDK9+MRE11 induces DNA damage. A) 22RV1 cells were treated for 24 hours with increasing doses of CDK9 inhibitor AT7519 and analyzed using western blot to assess the effects on RNA Pol II phosphorylation and DNA damage marker p-H2AX. **B)** C4-2 cells were treated as indicated and western blot was used to detect the relative levels of markers of interest. **C)** Cells were treated as indicated for 4 hours and the relative levels of MYC mRNA relative to actin mRNA were measured using RT-qPCR. Data is representative of 2-3 biological replicates. **D)** 22RV1 cells were treated as indicated for 24 hours and analyzed using western blot to assess the effects on cell death marker cleaved PARP and DNA damage marker p-H2AX.

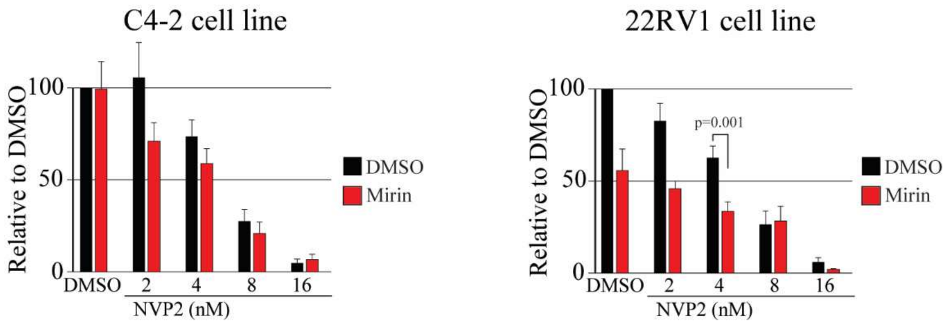

Supplementary Figure 6. Combined inhibition of CDK9 and MRE11 decreases proliferation of prostate cancer cells. C4-2 and 22RV1 cells were treated with increasing doses of NVP2 either in the presence or absence of MRE11 inhibitor Mirin at 10μM dose. The relative number of cells was measured using crystal violet-assay. The data presents an average of three biological replicates with SEM, and Student's t-test was used to evaluate the significance of the data.

Metastatic Prostate Adenocarcinoma (SU2C/PCF Dream Team)

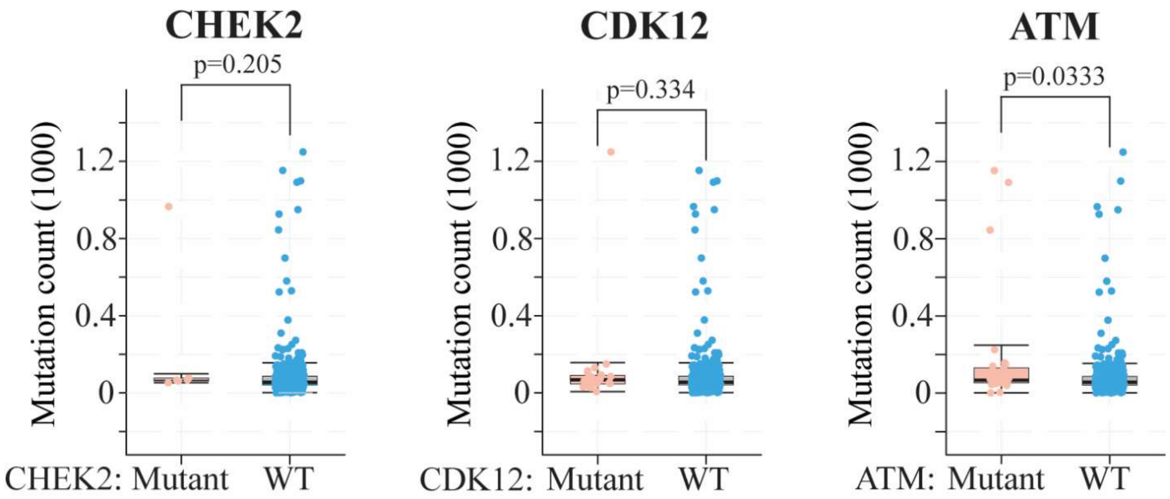

Supplementary Figure 7. Mutational count in the genomes of CHEK2, CDK12 and ATM mutant metastatic prostate cancer tumors. Bar plots depicting mutational burden. The plot was generated using cBioPortal and dataset Abida *& al.* (2019) [1].

References:

1. Abida W., Cyrta J., Heller G., Prandi D., Armenia J., Coleman I., Cieslik M., Benelli M., Robinson D., Van Allen E.M., Sboner A., Fedrizzi T., Mosquera J.M., Robinson B.D., De Sarkar N., Kunju L.P., Tomlins S., Wu Y.M., Nava Rodrigues D., Loda M., Gopalan A., Reuter V.E., Pritchard C.C., Mateo J., Bianchini D., Miranda S., Carreira S., Rescigno P., Filipenko J., Vinson J., Montgomery R.B., Beltran H., Heath E.I., Scher H.I., Kantoff P.W., Taplin M.E., Schultz N., deBono J.S., Demichelis F., Nelson P.S., Rubin M.A., Chinnaiyan A.M. and Sawyers C.L. Genomic correlates of clinical outcome in advanced prostate cancer. Proc Natl Acad Sci U S A 116(23):11428-11436, 2019.
2. Kuleshov M.V., Jones M.R., Rouillard A.D., Fernandez N.F., Duan Q., Wang Z., Koplev S., Jenkins S.L., Jagodnik K.M., Lachmann A., McDermott M.G., Monteiro C.D., Gundersen G.W. and Ma'ayan A. Enrichr: a comprehensive gene set enrichment analysis web server 2016 update. Nucleic Acids Res 44(W1):W90-97, 2016.
